# Multimodal repair in the semiotic landscape for social and political commentary

**DOI:** 10.1177/26349795261449663

**Published:** 2026-05-12

**Authors:** Amir Hossein Firoozkohi, Richard Feddersen, Grit Liebscher, Jennifer Dailey-O’Cain

**Affiliations:** 1 Department of Modern Languages & Cultural Studies, 3158 University of Alberta, Edmonton, Alberta, Canada; 2 Centre of Excellence for German and Arabic Language Studies, 68791 Ain Shams University, Cairo, Egypt; 3 Department of Germanic & Slavic Studies, 8430 University of Waterloo, Waterloo, Ontario, Canada

**Keywords:** written interaction, repair, semiotic landscapes, geosemiotics, ELLA, sociopolitical commentary

## Abstract

In spoken interactions, repair–the process by which interactants identify and resolve communication trouble–has been studied extensively. Fewer studies have explored the inherently multimodal repair process in written interactions, and almost none have examined it within the semiotic landscape, that is, on publicly visible signs. From a corpus of around 5500 images collected in Germany and Iran, we present a qualitative analysis of six selected cases to examine the functions of multimodal repair in social and political commentary. Using ethnographic linguistic landscape analysis (ELLA) and geosemiotics, the analysis focuses on images from Mannheim, Cottbus, Leipzig, Tehran, and Zanjan. Results indicate that actors in the semiotic landscape use different multimodal repair strategies for functions that go beyond identifying trouble in communication. Instead, they rely on repair strategies that are recognizable to viewers, but use these strategies to perform social and political commentary in different sociopolitical contexts. Implications of these findings for theories of multimodality are considered, especially in showing that repair is not fundamentally temporal but is shaped by the multimodal affordances (such as spatial organization) of the communicative context in which it occurs.

## Introduction

Research on semiotic landscapes (SL)^1^, or publicly visible communication using writing, images and other semiotic resources, has shown that the subtleties of written interaction in the material spaces where we coexist may go unnoticed in their profound interactional impact, despite being literally right before our eyes. As previous research has shown, these interactions can involve different actors “conversing” by using the walls, signs, and posts as the space for their interaction, often with one actor commenting on, obscuring, or obliterating a pre-existing message in processes that have been identified as a type of interaction (Feddersen et al., 2024). For example, Backhaus (2007) has shown that the addition of new layers to an old sign may lead to the communicative coexistence of older and newer versions, and Feddersen et al. (2024) have argued that such layering is governed by turn-taking mechanisms similar to those investigated by interactional linguists for face-to-face communication. Furthermore, even when multiple discernible turns are absent, the semiotic landscape is nonetheless inherently interactive by virtue of the fact that any semiotic display is viewed and interpreted by others (Feddersen et al., 2024: 50-51). As a result, the anticipation of future reactions is utilized in the design of any communication within the semiotic landscape. In light of these findings, we discuss the use of *repair,* the fundamental process through which interactants identify and resolve communication problems in both spoken and written interaction. We show how repair may be leveraged for social and political commentary in public asynchronous written interactions, leaving a potentially enduring impact due to the sustained visibility of these interactions.

Originally, repair was defined by interactional linguists as the process by which interactants address trouble in speaking, hearing, or understanding (Schegloff et al., 1977). Repair has been examined as a universally shared practice across languages and various forms of communication, primarily in face-to-face and telephone interactions, but also in written contexts, such as classroom discourse and social media exchanges (see section on repair in spoken and written interactions below). In this paper, we show some of the multimodal ways in which the repair mechanism is used in different kinds of SL contexts. Based on data from contexts as different as Iran and Germany, we argue in this paper both that aspects of the repair mechanism described for other spoken and written modes of communication can be effectively used by actors in the SL to negotiate sociopolitical commentary and that the implications of these findings should be considered in theories of multimodal interaction.

### Semiotic landscape and transgression

Semiotic landscapes (SL)—originally conceptualized as linguistic landscapes (LL) (Landry and Bourhis, 1997)—emerged within sociolinguistics to examine written language in public spaces, highlighting the visibility of language policies, multilingualism, and minority languages. As the field expanded to include issues of social justice, socio-political contexts, and identity (Bolton et al., 2020), scholars such as Shohamy and Waksman (2009: 314) offered a broader definition of semiotic landscapes as “verbal texts, images, objects, placements in time and space as well as human beings”.

This definition also brings into focus the aspect of *placement* in the SL, particularly as connected to *transgression*. In the SL, placement, that is, the physical context of a sign within the material world, shapes and generates meaning through the interplay of semiotic codes with place norms. Placement may also determine the sign’s degree of transgressiveness, understood as defying rules for the appropriate placement of signs (Bruyèl-Olmedo, 2025; Scollon and Scollon, 2003). Bruyèl-Olmedo and Juan-Garau (2022) examined linguistic transgression in Spain’s semiotic landscape, highlighting how various techniques, such as code erasure, exoticization, re-representation, and re-signification, were deliberately used to offer social commentary. These strategies served to assert identity, promote ideologies, challenge language policies, or secure commercial advantage by enhancing the visibility of shop signs. However, understanding transgression as “defying rules” (in terms of both placement and linguistic norms) is not always clear-cut. In fact, the concept of transgression has been shown to be more nuanced, in that deliberate acts of transgression “not only challenge the boundaries and mechanisms that sustain categories and ways of thinking but also produce other ways of thinking” (Pennycook, 2006: 41). Similarly, Pavlenko (2009) observed that different sets of norms can be at odds and that transgressiveness cannot always be determined.

Traditionally, transgressiveness in the SL has been defined reductively in terms of violating norms. However, by expanding on the above argument, we will show some additional ways in which transgressing in the SL can transcend this kind of normative perspective. As Bruyèl-Olmedo (2025: 156) writes: “when we deprive our urge to ‘go beyond’ from negative preconceptions, transgression embodies the dialogue between social complacency and our questioning of the norm, but also between the transgressing ‘self’ and the social ‘other’”. Communicatively speaking, “transgressing” becomes broader than simple norm-violation under this interpretation, encompassing acts that serve the societal function of civil courage.

This understanding of “transgression” as acts of civil courage aligns with Martín Rojo’s (2016: 49) argument that “a new understanding of politics, citizenship and the new economy” may emerge with a change of discourses in the semiotic landscape that largely involve processes of deterritorialization and reterritorialization. Such processes lead to changes in the conditions of production and circulation of communicative practices. Research investigating such practices seeks to provide new ways of understanding by challenging conventional spatial organization and uses and by rearticulating them as expressions of alternative beliefs and rituals, which is very much in line with our focus as well.

### Repair in spoken and written interaction

Schegloff et al. (1977: 363) argue that what counts as a trouble source or repairable item is a participant’s construct; consequently, even propositionally “correct” utterances may be treated as problematic when they involve “trouble in speaking, hearing or understanding” (ibid.). They further show that repair in conversation is used systematically (see also Raymond and Sidnell, 2014), including who initiates and completes the repair (self or other), its placement relative to the trouble source (same turn, transition space, next turn, or later positions), and the identification of the trouble source. Extending this work, Schegloff (2013) identifies 10 operations of self-initiated, same-turn repair—replacing, inserting, deleting, searching, parenthesizing, aborting, sequence-jumping, recycling, reformatting, and reordering. Although developed for talk-in-interaction, these operations inform our analysis in the written medium of SL, which also draws on research on repair in written interaction, discussed next.

While research on repair in written contexts is still relatively limited compared to repair in spoken interaction, exceptions include research on chat-based conversations (Collister, 2011; Meredith and Stokoe, 2014; Tudini, 2016) and email communications (Tanskanen and Karhukorpi, 2008). Within the context of chat conversations, Schönfeldt and Golato (2003) observed that participants utilize repair initiation strategies from talk-in-interaction and apply them to written contexts. Research on repair in educational settings, particularly second language learning, is also relevant for our analysis. Studies show how repair in such contexts can be interactive, but also shed light on the multimodal resources interactants use: Second language learners engage in repair practices during collaborative writing tasks (see Balaman, 2021), and teachers and learners use corrective feedback strategies (Ellis, 2008) to address and resolve errors in essay writing (see Olatunji et al., 2023).

The shift from spoken to written interaction means a shift in multimodal resources. For example, repair in spoken conversation relies on a temporal sequentiality that is often not observable in documents or photos of written interactions. However, written repair is still sequential, but relies on spatial organization (e.g., Kress and Van Leeuwen, 2020). Other aspects of temporal organization in spoken conversation also have equivalents in written interaction. As our analysis will show, crossing out or writing over text may correspond to overlaps in spoken interaction, for instance.

Overall, these studies illustrate the adaptability of the repair mechanism, which interactants naturally extend and apply to various communicative modes and contexts. The cited research also shows how helpful the fine-grained conversation analytical tools are for the analysis of data not traditionally considered within the field of SL. Our study further contributes to this line of inquiry by applying the concept of repair to interaction in the SL as another written, multimodal, and interactive context. In our focus on the socio-political dimensions of meaning-making in the SL, we also see ourselves contributing to a line of interactional research interested in uncovering the use of repair to negotiate and contest socio-political aspects. Arguably, this line of research may be traced back to Jefferson’s (1974) ground-breaking research on the negotiation of identities through repair. As Jefferson (1974) skillfully demonstrates, interactants may use fine-grained details of linguistic repertoires to critique the social order, while eluding consequences by allowing several ways of understanding the utterance. This recalls Bakhtin’s (1981, 1986) dialogic view, which sees language use relying on and “echoing” previous uses of an utterance, including “double-voiced discourse” (ibid.). Drawing on Bakhtin then allows us to account for the different “voices” that may manifest in any language use, and that attach meaning to an utterance, often based on identities and ideologies. To reveal how these meanings are created, based on the use of the repair mechanism, is the goal of this paper.

## Methodological framework: Data and analysis

Our methodological framework is anchored in the principles of geosemiotics (Scollon and Scollon, 2003) and ethnographic linguistic landscape analysis (ELLA), introduced and developed by Blommaert (2013). These complementary approaches have guided our data collection in the SL as well as our analysis.

Building on geosemiotics, we start with an understanding that the meaning of a sign is derived from its “where” and “how” of placement in the material world. By integrating information about interaction order, visual semiotics, and place semiotics, geosemiotics leads us to examine how meaning is created by ways in which social actors organize interactions, and the discourse of texts and images in a particular time and place (Scollon and Scollon, 2003:166). ELLA allows us to transcend the static nature of signs to understand their social functions and evolutionary trajectories by considering three axes: the historicity of situated human action (past), intended addresses and prolepsis (future), and the sign’s physical placement and its relation to other signs (present) (Blommaert and Maly, 2015; Maly, 2016). Employing these frameworks in our discussion of repair in the SL allows us to understand its socio-political effects.

We focused our analysis on two collections of public images, one from Iran and one from Germany. The Iran data encompasses 400 images collected by Mohebbi and Firoozkohi (2021) as part of a study on the typology of errors in the use of English in Iran in 2018, and 822 pictures of political protest signs in the SL of Iran, as part of Firoozkohi's ongoing dissertation collected from social media and through personal contacts in Iran between September 2022 and June 2024, amidst the Woman-Life-Freedom (WLF) Movement. The data from Germany stems from a collection of 5000 photos taken in the German cities of Leipzig and Mannheim between 2019 and 2021 as part of a larger project. Finally, we added individual photos taken in the city of Cottbus, Germany, by one of the authors who resided there in 2023.

We chose to include examples from two such different countries to show similar repair strategies despite drastically different sociopolitical contexts. Drawing on Bakhtinian dialogism (cf. Bakhtinian dialogism, 2014), we examine different datasets which can combine and create a single, coherent line of argument that carries multiple voices or perspectives at once and expands our conceptual horizon of the mechanism of repair in the SL.

Based on research on repair in talk-in-interaction, we first defined repair in the SL as a deliberate semiotic act that is interpretable as targeting a trouble source, and, in the process, modifies the meaning of a pre-existing semiotic display. Next, we individually examined the datasets with which we were most socio-politically familiar and coded four to six examples in each dataset that aligned with this definition^2^. We then discussed and excluded those examples that did not meet our established criteria. We found that in some cases, the source of trouble (i.e., the reason for the repair) seemed to have sociopolitical motivations. Consequently, we focused on these cases and revised our understanding of repair and its sociopolitical implications, based on emergent patterns and a stringent analysis guided by geosemiotics and ELLA, as discussed above. Going through the images together as a research team allowed us to utilize our respective (un)familiarity with the relevant sociopolitical contexts and to draw on outsider perspectives (see Collins and Slembrouck, 2007), as one author was familiar with Iran but not Germany, and three authors were familiar with Germany but not Iran.

**Figure 1.**
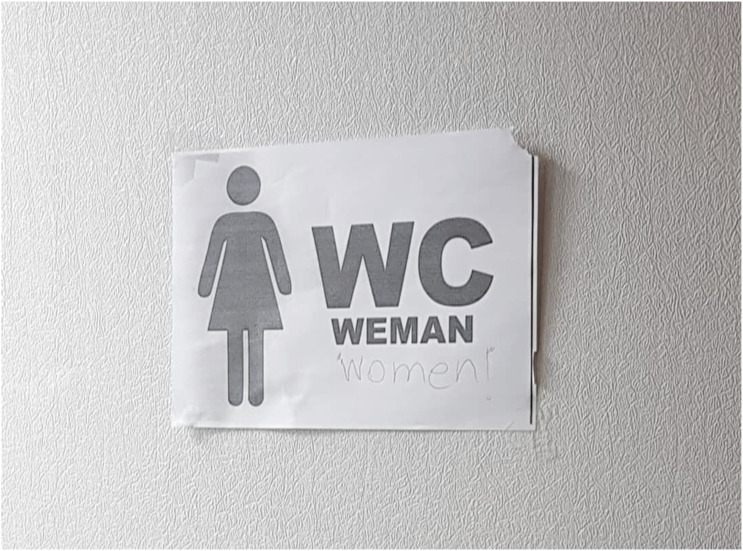
Instructive correction in the SL of Tehran, Iran.

**Figure 2.**
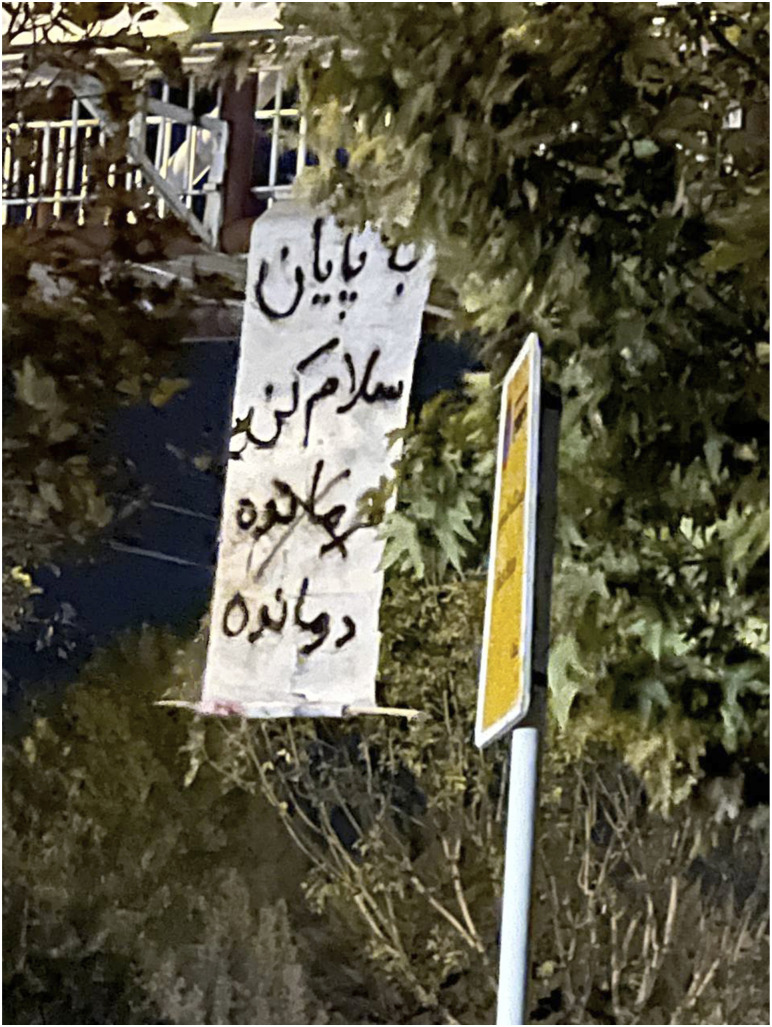
Concessive repair for political defiance in the SL of Zanjan, Iran.

**Figure 3.**
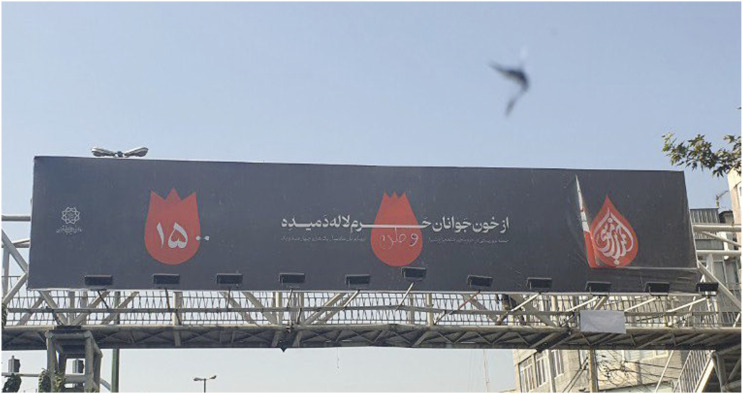
Replacing and inserting repair for contesting ideologies in the SL of Tehran, Iran.

**Figure 4.**
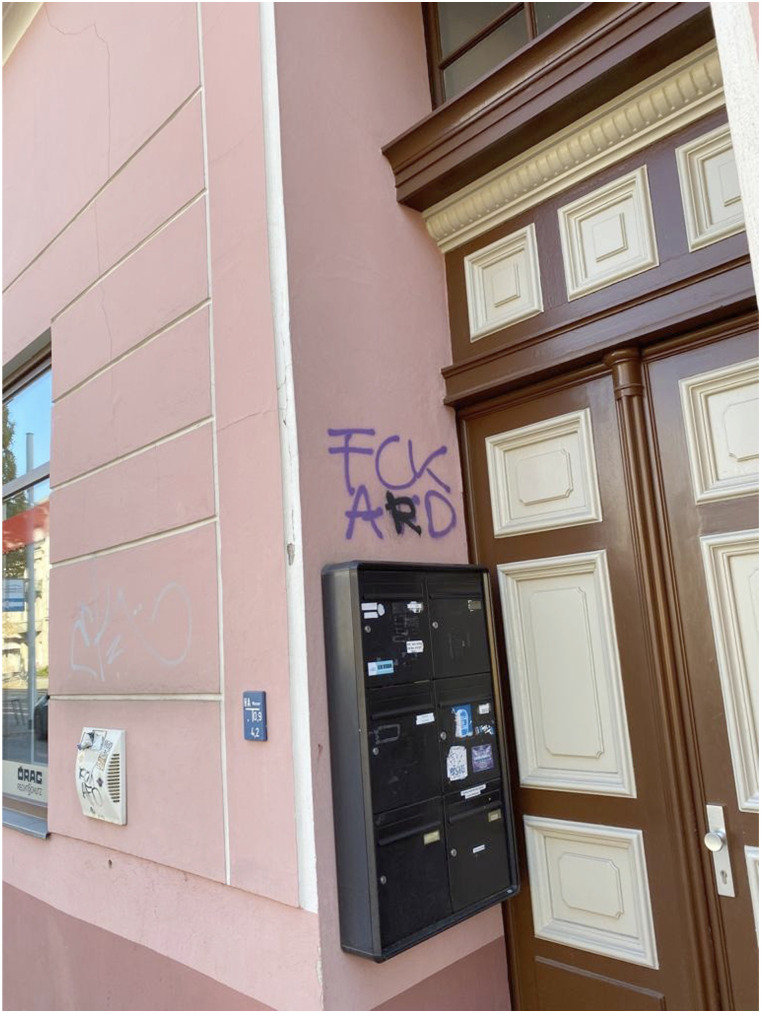
Recycling repair for contesting ideologies in the SL of Cottbus, Germany.

**Figure 5.**
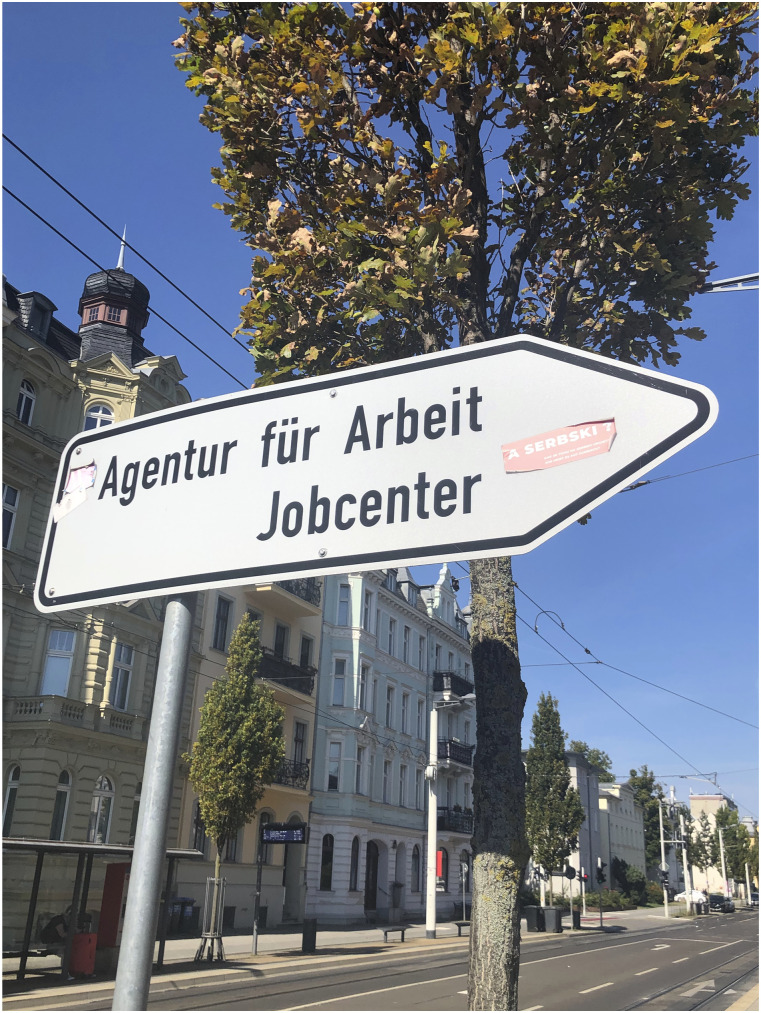
Defacing repair for highlighting minority language policy issues in the SL of Cottbus, Germany.

**Figure 6.**
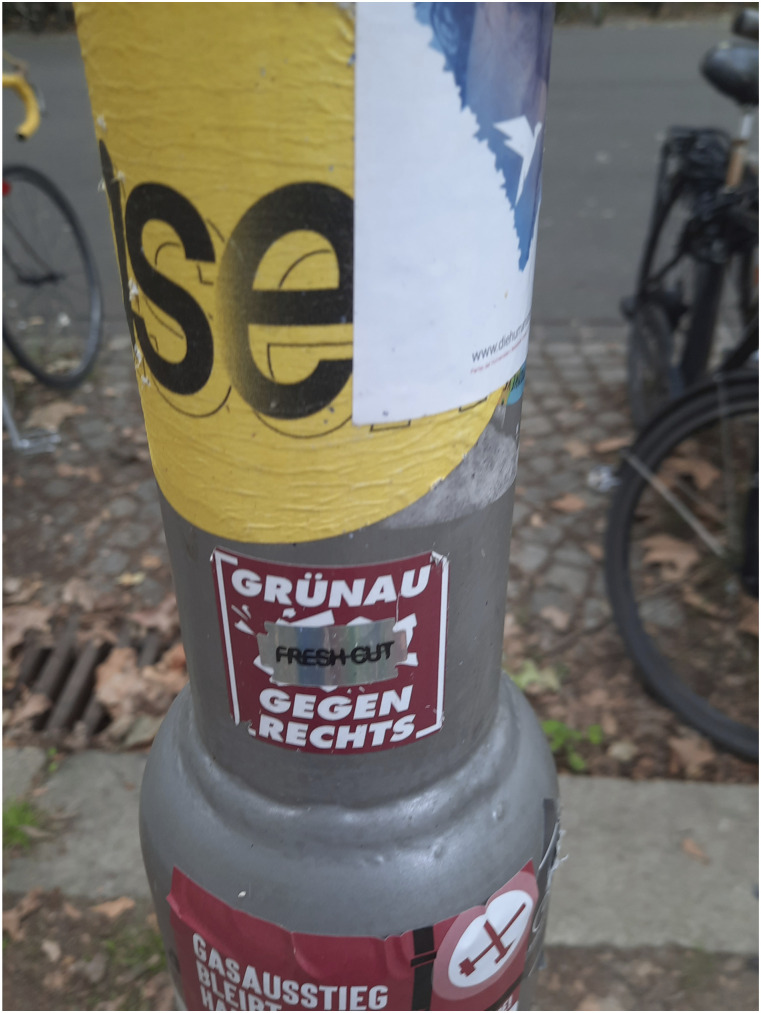
Placing repair for censoring and adjusting political messages in the SL of Leipzig, Germany.

For our analysis section below, we selected six representative examples (Figures 1 through 6) that demonstrate how different repair strategies are used for social and political commentary in the SL. We show that several repair operations are borrowed from conversational and written repair (Figures 1 to 4), with additional multimodal repair strategies that are exclusively relevant to the SL (Figures 5 and 6).

## Analysis

The following six examples demonstrate how different repair strategies are used to provide different kinds of sociopolitical commentary. We start with an example that, superficially, appears to be the correction of a spelling mistake. Considering the concept of voice (see above), Figure 1 also contains some social critique that stems from its particular placement. We will then move on to examples of repair in which the critique is more explicit as a result of social and political issues targeted as the main sources of trouble.

Figure 1 depicts a sign for a women’s washroom in a university in Tehran, featuring an English-language misspelling (“weman”). The way the paper is taped to the wall or door, with visible creases and tears, and the casual approach of using print on paper rather than an engraved metal plaque, indexes a non-permanent solution to a signage need.

Below the erroneous form, the word “women” is correctly spelled with a black pen. Though no corrective markers are used (such as crossing out the misspelled word), the placement right underneath “weman” suggests that “weman” is made the target or trouble source. Consequently, “women” is the suggested alternative, making this a specific kind of repair commonly identified as an “instructive correction” (see Bolden, 2024). As we can see, the spatial “below” here corresponds to the temporal “after” in spoken conversation, which means it occurs in a transition space rather than an overlap. In other words, the blank space beneath the misspelled word as an affordance corresponds to a *transition relevance place* (TRP) in face-to-face interaction—a point of possible completion that cues the other participant to take their turn (see Liddicoat, 2022).

While any correction can be done by the self (i.e., the author), evidence from the particulars of the writing suggests that this is an other-correction rather than a self-correction, which then further results in a social critique. The other-correction (and social critique) is also evident, as the word “weman” is appended with an exclamation mark and quotation marks. Exclamations, in written communication, convey intense emotional and affective attitudes, with either positive or negative effects (Collins, 2005; Leech, 2007). In Figure 1, the exclamation mark suggests the repair initiator’s surprise or annoyance (see Bouazizi and Otsuki Ohtsuki, 2016; Unger, 2019) at the misspelling of a common word. The exclamation mark can then be read as demanding to pay attention to the correct spelling, resembling a gesture of a raised eyebrow or a pointed index finger in face-to-face communication, assuming that the use of exclamation marks and punctuation, in general, can carry meaning as prosody and gesture do (Bavelas and Chovil, 2018; Sanchez-Stockhammer, 2016). Similarly, the emphatic quotation marks around “women” emphasize the correct form. This interpretation aligns with sociolinguistic and semiotic considerations regarding the functions of quotation marks beyond their citational purposes (Abbott, 2003).

In addition, the absence of a strike-through in Figure 1 is meaningful. Crossing something out, similar to using asterisks as a discourse marker in written communication (Collister, 2011), is considered a common corrective feedback strategy for contesting what might be erroneous. Moreover, parallel to overlapping speech in spoken interaction (see Lerner, 1996), crossing out could be perceived as intrusive. By leaving the incorrect form unmarked without striking it through or changing the letters “e” to “o” and “a” to “e”, the repair initiator managed to keep the original (incorrect) form visible for the benefit of “non co-present others” (see Bolden, 2024: 202), including students and academic or non-academic staff members, to notice and compare the forms. In summary, Figure 1 shows an error correction where the repair initiator draws on their “epistemics of expertise”—that is, epistemic authority grounded in subject-matter, professional, or linguistic expertise—to intervene and offer a repair solution (Bolden, 2013: 331). The academic context, as the background to the correction, further makes this a form of social critique, suggesting that such a misspelling is inappropriate in this context. The transgressivity of repair in this example derives from the uninvited repair solution to the trouble source by a co-present other who may not have as much institutional authority as the sign producer, a difference made visible through the use of handwritten repair as opposed to the printed original text. Notably, it is the multimodality of these spatial affordances that allows the repair work to be interpreted as transgressive.

The following two examples, taken from political protest signs in Iran during the 2022 WLF Movement, illustrate three repair strategies, namely, concessive, replacing, and inserting repair (see Couper-Kuhlen and Thompson, 2005; Schegloff, 2013), used to undermine authority figures and reclaim a sense of patriotic identity, respectively.

The hand-written banner, captured on October 30^th^, 2022, hangs from a footbridge in Zanjan, a northwestern city in Iran. The following Interlinear Morphological Glossing (Lehmann, 2004) in Table 1 represents the writing on the banner in Figure 2 to illustrate the function of each morph. The first column on the left presents the original Farsi words displayed on the banner. The second column provides their Finglish equivalents (i.e., Farsi written in the Latin script). The third column offers a word-for-word translation, and the final column presents a free translation of the sentence.

**Table 1.** Interlinear morphological glossing of Figure 2.

1	به پایان	Be payan	To the end	Say hello
2	سلام کن	salam kon	say hello	To the end
3	فرمانده	farmandeh	commander	commander
4	درمانده	darmandeh	miserable	miserable

We argue that sociopolitical commentary in this example is achieved through a specific type of repair, known as *concessive repair* in face-to-face interaction, which involves self-repair of statements that challenge one’s belief systems or overstatements that might result in false assumptions and disagreement (Couper-Kuhlen and Thompson, 2005). Here, the structure of concessive repair, which is familiar to the interactants from face-to-face interaction, combines with language play (which will also be discussed in Figure 4) to produce a political message. The repair involves three stages: overstatement, concession, and revised statement.

The initial semiotic turn is the line translated as “say hello to the end, commander”^3^. Its meaning is deeply embedded in Iran’s sociopolitical context. The writing on the banner ironically uses part of an epic song, *Hello Commander,* which is themed around generational support for the Islamic Revolution. In 2020, this song was widely broadcast and promoted by the Islamic Republic’s state media. Although the producers of this song declared that the term “commander” refers to the twelfth Imam of Shias and that the young generation pledges allegiance to him, grassroots activists in Iran believed that the song was a laudatory attempt to pay homage and promote a strong and positive public image of the political leader, who is also the Commander-in-Chief of the Armed Forces. On social media, particularly on X and Instagram, the public likened the song to the brainwashing attempts of the Nazi era (Sinaiee, 2022), when music served as a medium for ideological conversion, propaganda, and the inculcation of Nazi beliefs (Neuschwander, 2012). The dissenters also posted social commentary on various platforms with alterations to the original song, using derogatory words such as “darmandeh” (*miserable*) or “pasmandeh” (*waste*), which rhymes with “farmandeh” (*commande*r). Language play and rhyming words have been extensively employed in verbal and written sociopolitical slogans across various contexts as discursive strategies to evoke powerful effects and attract attention (see Romano, 2021; Van De Velde, 2024).

In line three on the banner, “commander” (the overstatement) was targeted as the trouble source, deliberately struck through (the concession), and replaced by the term “miserable” (the revised statement). Each of these steps was carried out *before* hanging the banner, and by the same individual(s) writing the original message, which is evident from the neat spacing between words and their relation to the length of the banner, the apparent continuity between writing utensils, as well as the uniformity of the handwriting. The author also created their own transition space by leaving room at the bottom for the repair, another example of how sequentiality is indicated spatially. By creating a message of defiance and revolution, this banner may suggest that the opposition rejects the commander’s leadership. The change from “farmandeh” (*commander*) to “darmandeh” (*miserable*) exemplifies Bakhtin’s notion of vari-directional double-voiced discourse, wherein the second voice—such as in parody and other forms of humour—is used for criticism, ridicule, or attack (Baxter, 2014). This is similar to “offensive humour” in political contexts, which allows marginalized and underrepresented groups to be heard and to make social commentary (see Graefer et al., 2019). The result is a message that uses the structure of a conventional self-repair not to simply present a corrected version of the banner, but to create a message of defiance and revolution in opposition to the power structure.

The next example of Figure 3 features a state-sponsored billboard designed following the ISIS terrorist attack on the Shah Cheragh Shrine in Shiraz, Iran, amidst the WLF Movement. It displays contesting ideologies and uses the repair strategies of *replacing* (Kitzinger, 2012; Schegloff, 2013) and *inserting* (Schegloff, 2013) to reclaim public space.

The following Interlinear Morphological Glossing in Table 2 represents the writing in the middle part of Figure 3.

**Table 2.** Interlinear morphological glossing of Figure 3.

1	دَمیدِه	لاله	حَـــــــرم	جَوٰانان	خون	از
1.1			وطن			
2	damideh	laleh	haram	Javanan-e	khune	Az
2.1			vatan			
3	From	the blood	of the youth	of the shrine	tulips	have sprouted
3.1				the homeland		
4	Tulips	have sprouted	from	the blood	of the youth	of the shrine
4.1						the homeland

The main message in the middle of this billboard was adapted from the seventh *tasnif* (a literary genre similar to a ballad) by Aref Qazvini, a poetic tribute to those who sacrificed their lives for freedom during the Constitutional Revolution of 1905–1911. It draws upon the mythological narrative of Siavash in Shahnameh, where tulips are said to have sprouted from his spilled blood. Throughout history, this message has resonated with revolutionaries and has been adapted in pivotal moments, such as the 1979 revolution and subsequent unfinished social movements in Iran, including the WLF Movement.

In the spirit of political protest, the protestors attempt to reclaim and redefine the significance of the original message in Figure 3, and this is where the repair is initiated. By writing the word “the homeland” beneath “shrine”, they redirected the focus of the message, emphasizing the broader struggle for liberty. Additionally, the number of casualties of the terrorist attack, “15” (۱۵), adorning the tulip on the left, was altered to “1500” (۱۵٠٠) by adding two zeros to the number, which is a reference to the significant casualties during the November 2017 protests. This repair work, which uses replacing and inserting operations (Kitzinger, 2012; Schegloff, 2013; Wilkinson and Weatherall, 2011), serves to communicate a political message by underscoring the protestors’ prioritization of ethnonationalism and homeland over Shiite nationalism—which is represented by the word “shrine” in the main text and the name “Ahmad ibn Musa”^4^ written in calligraphy on the right-hand side. Moreover, in high-quality printing of sacred texts and poetry such as the Quran or the Divān of Hafez, and mosque calligraphy designs, diacritic signs and *Kashide*^5^ are typically used to indicate proper pronunciation, add emphasis and beauty, justify texts, and improve legibility (see Azmi and Alsaiari, 2014; Berry, 1999). On this banner, the Kashide seems to ironically reference this use, potentially highlighting the state’s religious nationalism and acknowledging its deep bond with Islam.

In this example, the act of repair resonates with Bakhtin’s multiplicity of voices, as the initiator of repair chooses a dialogic backdrop to reframe the trouble source, just as a polemicist employs one to twist the quotation of an opponent (see Bakhtin, 1981: 340). Consequently, we conclude that the resemiotization of messages here represents a recurring dynamic in conflict-affected societies, where opposing forces engage in a tug-of-war over symbols and slogans, repurposing them to serve their respective narratives and agendas.

In the remainder of our examples, which come from Germany, we will present three further repair strategies used for social commentary. Similar to Figures 2 and 3, the example in Figure 4 borrows from conversational repair operations and draws on multimodal resources.

The photo in Figure 4, taken in the city of Cottbus, shows graffiti on the entrance of a residential building. Similar graffiti using these letters can be found throughout the city, with some texts displaying the original message “FCK AFD” and others “FCK ARD”. The presumably first graffiti in this case, written in purple spray paint, reads “FCK AFD”^6^, referring to the German right-wing extremist party *AfD* (*Alternative für Deutschland*, Alternative for Germany). The “F” in “AFD” has been written over with noticeably thicker black spray paint to obscure the original letter and replace it with an “R, turning the phrase into “FCK ARD”, referring to the ARD, a public (that is, neither privately owned nor government-run) broadcasting organization. This is another example of how temporal sequentiality in spoken conversation becomes spatial sequentiality in written interaction. By writing over existing text, an effect akin to overlapping speech is achieved.

We argue that this modification is a form of social commentary rooted in the political factions both versions represent. While “AFD” is straightforward as it directly refers to a political party, understanding why this has been changed to “ARD” requires knowledge of the enduring attacks on “mainstream” media by the members of the far right. The term “Lügenpresse” (lying press), similar in meaning to “fake news”, has a long history and was used by the Nazis then and now to delegitimize free journalism. The first turn in this photo, therefore, represents a left-wing or antifascist stance, which was corrected to represent a right-wing or fascist stance. The potential for meaning-making is provided through repair.

This example combines another repair operation—*recycling* (Schegloff, 2013), a type of self-initiated, same-turn conversational repair—with the replacing operation discussed in the previous example. Recycling typically involves the speaker repeating a segment of speech shorter than a full TCU. According to Schegloff (2013), while recycling serves as a means to an end achieved through other repair operations, it is worth considering as an operation in its own right. In this case, the replacing operation is framed by the recycled elements “A” and “D” in “FCK ARD”, as well as the shape and size of “F” from the previous turn, which together guide the repair work toward “R”.

In the next two examples, Figures 5 and 6 illustrate multimodal repair strategies unique to the SL context, which we call *defacing* and *placing* repair, adapted from Schegloff’s categorization. These strategies are used in these data, respectively, to reframe a political message, criticize German language hegemony and conceal an offensive symbol.

In each of the previous examples, we could identify both repair initiation and repair completion. In the following example (Figure 5), the repair is initiated but not completed on the sign itself, but implied or requested, which is at the centerpiece of the socio-political critique. The photograph in Figure 5 was taken in the city of Cottbus, Germany, near the Polish border, and the cultural centre of the Lower Sorbs, an indigenous Slavic minority group within Germany. The Upper and Lower Sorbian people’s rights to the protection of their national identity, language, religion, and culture are enshrined in the constitutions of Germany’s states of Brandenburg and Saxony. Since 1994, the law regarding the manifestation of the Sorbs’ rights in the state of Brandenburg stipulates which public signs and signs indicating buildings of public significance in the Sorbs’ ancestral settlement region are to be written in both German and Lower Sorbian^7^.

In 2023, by no means were all or even most of these signs in Cottbus bilingual, except for street names. In February 2022, several Lower Sorbian signs in Cottbus were covered in graffiti or with stickers, which the Sorbian governing body, Domowina, considered a deliberate attack on bilingualism in Cottbus^8^. In response, several stickers printed in batches, as seen in Figure 5 below, were placed by unknown individuals on monolingual German signs in Cottbus. The repair in this example arises from a political motive related to Sorbian language ideologies and ethnolinguistic vitality.

The photo in Figure 5 shows a white metal sign with a black frame in the shape of an arrow pointing to the right. In the centre of the sign, “*Agentur für Arbeit*” (agency for work) and “*Jobcenter*” are written. The original sign is marked up with stickers (i.e., defacement repair). In the top-left corner of the sign, there are faded, unintelligible stickers. On the right, which is our focus, there is a sun-faded red sticker that reads “A SERBSKI?” (And Sorbian?), and smaller, but still in capital letters below that: “KAK SE TOMU NA SERBSKI GRONI?”, with the German translation at the bottom: “WIE HEIẞT ES AUF SORBISCH?” (What does it mean in Sorbian?).

We can draw parallels between the repair mechanism in this semiotic display, indirect corrective feedback provided in language classroom contexts, and open-class repair initiation in spoken interactions. On the one hand, the “A Serbski?” sticker, placed in the margin of the sign but not on top of the original writing, indirectly indicates an error in its proximity, comparable to indirect corrective feedback, which instigates the correction (i.e., adding Sorbian to the sign) as a request. This indirect mention of the absence of Sorbian might make a more profound impact than the previous aggressive vandalism of monolingual German signs. In comparison to a previous covering of Sorbian signs with soccer and Hooligan stickers, this approach comes across as less aggressive, possibly also because it uses the indirect corrective feedback strategy (elicitation), familiar to all viewers from instructional contexts, to rephrase the utterance in Sorbian.

On the other hand, the sticker resembles open-class initiators in other-initiated conversational repair, in which question words point to the detection of some trouble in the previous turn (see Sidnell, 2010: 117). Therefore, the question on the sticker does something beyond posing a simple question (see Schegloff, 2007: 75) to instigate a repair proper, similar to what the exclamation mark in Figure 1 does. For example, Dersley and Wootton (2001) observed that wh-questions can be posed to complain. In addition, they can also be used to challenge previous utterances (see Koshik, 2003). Similarly, by asking “A SERBSKI?” the repair initiator(s) protest about the exclusion of Lower Sorbian and make reference to a neglected language policy. Their assumed aim may also extend beyond the desire for bilingual signage and may refer to the overall status of the Lower Sorbian language in society. As Martín Rojo (2016: 58) argues in her analysis of the Spanish Indignados movement, these semiotic resources “function as a text designed to stimulate action or, at least, to provoke reflection on the current state of society”. Therefore, the sticker can be understood as a form of double-voiced discourse that also resonates with Fairclough’s (2009) notion of “power to,” whereby each conversational turn opens up the potential to produce behavioural or material change through the illocutionary force of speech acts (Baxter, 2014).

By the same token, exclusionary policies aimed at underrepresenting minority languages in a region can lead to campaigns and graffiti movements as a call to action (see Landry and Bourhis, 1997; Vuorsola, 2020). In the SL, the person placing the sticker is interacting with both the state responsible for placing public signs and the broader public witnessing the exchange. The public attention seems to be the goal here, that is, to make the state aware of the issue, since the repair initiator could have written a formal letter to the responsible entities instead. The sticker is therefore also a response to the prior vandalism of bilingual signs in Cottbus. By responding to the erasure of the Sorbian language on signs with this sticker that assumes sufficient knowledge and resources to add Sorbian, the interactant emphasizes the place Sorbian is owed in the SL.

Overall, we argue that the repair initiation in Figure 5 can address not only troubles in speaking and understanding but also issues within broader social discourses, without necessarily reaching a resolution or completion.

Our last example, shown in Figure 6, is equally political but unique in its way of simultaneously addressing the source of trouble and the trouble source. In addition, the repair is achieved not through linguistic means but by using multimodality in layering stickers on top of each other to literally and figuratively censor a hate symbol.

Figure 6 shows a photo discussed previously in Feddersen et al. (2024: 35-36) as an example of the interactive SL. We are returning to it here because it shows a creative multimodal way to use the repair mechanism for a political message. In particular, we want to argue that the image contains a repair in the SL that achieves two things: it adjusts the original message, and the adjustment addresses a larger socio-political context.

The photograph shows a sticker that appeared on a traffic light pole in Leipzig, Germany, in 2021. The sticker reads “Grünau gegen rechts*”* (‘Grünau against the right’), where Grünau refers to an outer-suburban neighbourhood of Leipzig. On top of this original sticker, and right in the centre, someone placed a smaller second sticker shaped like a razor blade with the words “FRESH CUT” written on it in English. As we have argued in Feddersen et al. (2024: 35-36), this second sticker, originally referencing a fashion label (Fresh Cut), seems to have been carefully placed on the first sticker so as not to obscure the text but only some picture that appeared in the middle of the first sticker. That picture is still recognizable as a fist breaking a swastika, especially to anyone familiar with similar images in the context of the political right.

While the picture of the first sticker itself indexes anti-Nazism, it is also controversial since displaying the swastika symbol in public places is largely illegal in Germany and is considered taboo in society, even in such anti-Nazism messages. The repair addresses the sociopolitical context in which a Nazi symbol is illegal as the source of trouble. The swastika is the trouble source here, and the image of the razor blade addresses or even symbolizes the action that it is promoting, supported through the language (i.e., cutting it out). Therefore, the careful placement of the razor blade makes this strategy unique to the SL and different from crossing out (Figures 2 and 3) or defacement (Figure 5). Its function is to censor a symbol considered to be hateful and harmful in society; the social critique targets the symbol without undermining the original message.

## Conclusion

In proceeding from the perspective that the SL is inherently interactive (Feddersen et al., 2024), we have shown that a multimodal repair mechanism structurally similar to that found in spoken interaction and/or other written contexts is also present in the SL. Furthermore, we have argued that these similarities are no accident, as interactants in the SL creatively adapt the existing repair mechanism that they are already familiar with from spoken or other written contexts. Interactants rely on repair from these other contexts because elements of repair fundamental to spoken interaction, such as the sequential order of conversation (as seen, for example, in overlapping speech), are absent in the SL. Doing repair work in the SL, therefore, requires social actors to adopt new, and fundamentally multimodal affordances available within the SL to carry out similar functions as in face-to-face repair.

This implies that multimodality is a crucial aspect of repair work in the SL. In repair work as originally studied within spoken interaction, the temporal sequential order of face-to-face conversation does the heavy lifting. This does not imply that repair is exclusively temporal in spoken interaction, as embodied-visual aspects of interaction, such as eye gaze or gesture, are of course an additional resource for repair work (Saalasti et al., 2023), simply that the temporal aspect is a fundamental one. In the use of repair in the SL, on the other hand, this heavy lifting is taken over by spatial organization and is inherently multimodal. Repair work in spoken conversation always relies on the temporality of the sequential order of turns; similarly, repair work in the SL always relies on its inherently multimodal spatial elements. Future research that might shed more light on this might involve studying viewers’ eye movements with an eye tracker while they are interpreting an instance of repair within the SL (as in Van der Sluis and Mellema, 2025). This could help us understand where people’s gaze falls during this interpretation, as well as which elements are interpreted first, second, third, etc. This research would also provide additional insights into how similar or different the spatiality of turns in the SL’s repair work is to the sequentiality of turns in repair work found in face-to-face interaction.

In the examples we analyzed, interactants then use these multimodal spatial elements, drawing on their knowledge of repair in other contexts. As we have argued, they do this to perform various types of social critique rather than addressing typical trouble in communicating, such as misunderstandings or misreadings. The social critique relies on several elements in order to be interpreted in this way: from a mutual knowledge of the way repair plays out in face-to-face interaction, from the social context in which the semiotic turns are physically placed, and, in some cases, the incomplete and open-ended nature of the repair (cf. Figure 5).

We find that the communication of this social critique is the primary message in each of our examples. This is the case even for examples like Figure 1 (the correction to “women”), which may superficially look like the direct application of a common corrective feedback strategy known from a classroom context. At a second glance, it becomes clear that the primary message is social critique, as the university location makes this a reprimand for making such a spelling error, rather than simply fixing a communication problem.

In Figure 2 through 6, the inherent sociopolitical commentary through repair is even more apparent. In particular, some trouble sources are targeted as repairable because the social and political context gives an additional impetus for intentional transgressive action (see Bruyèl-Olmedo, 2025). This explains the higher occurrence of social and political repair or commentary in the dataset, where intentional “transgressivity” becomes an important part of meaning-making, as in Figures 2 and 3. In both these Figures a crossing-out strategy is also employed to make small linguistic alterations to previous semiotic turns to make political points about Iran’s leader and the overall political situation during a period of social uprising. In Figure 5, while the repair mechanism superficially resembles a request for a translation to address trouble in comprehension, further analysis reveals that the core message is instead a demand to comply with the law and to honour the rights of an ethnic and linguistic minority group. Finally, in Figure 6, the covering effectively “corrects” not linguistic material but an offending political symbol while at the same time preserving the anti-racism message in the previous semiotic turn.

As stated above, in each of these cases, the similarity to the repair mechanism already known to all communicators, from either spoken interaction or written communicative contexts, or both, is intentional. In fact, the similarity to the repairing of communication trouble in these other contexts is crucial to communicating and carrying out the desired social commentary. In other words, the inherent repair mechanism needs to be recognizable to future viewers of the semiotic display because it is this very mechanism that allows the social meaning to emerge and be interpreted as such by those future viewers.

In sum, we have shown, across these different cultural contexts, that repair in the SL may perform a commentary with a social or political message at its core rather than addressing literal trouble in communication. This finding expands upon previous research on the inherently interactive nature of the SL, while also illustrating the flexibility inherent in the repair mechanism itself. In its adaptation to other contexts, such as the SL, repair still preserves and, in essence, relies on interactants’ knowledge of the mechanism from other contexts to achieve social and political commentary as its ultimate message.

## Data Availability

The datasets generated during and/or analyzed during the current study are not publicly available due to ethical and privacy considerations.

## Notes

1 While the terms ‘linguistic’ and ‘semiotic’ landscape are often used interchangeably in the field, we will use the latter to highlight its multimodal nature.

2 Using Atlas.ti in coding the German dataset proliferated our analysis.

3 Comma added by the researchers to make the meaning clearer to non-Farsi readers.

4 Ahmad ibn Musa, buried in the Shah Cheragh, is the son of Musa al-Kazim, the seventh imam in Twelver Shia Islam.

5 The elongated horizontal line between letters “ح” and “ر” in the word “حـــــرم”.

6 The letters “FCK” (a censored version of “fuck”) plus an acronym or word written without vowels, preferably also three letters long and in a second line underneath, is a common, recognizable meme-like format to express distaste for something.

7 See: Gesetz über die Ausgestaltung der Rechte der Sorben/Wenden im Land Brandenburg (Sorben/Wenden-Gesetz - SWG).

8 See: Angriff auf die Zweisprachigkeit.
